# The neural economics of brain aging

**DOI:** 10.1038/s41598-021-91621-5

**Published:** 2021-06-09

**Authors:** Jacob Kosyakovsky

**Affiliations:** University of Virginia School of Medicine, 200 Jeanette Lancaster Way, Charlottesville, VA 22903 USA

**Keywords:** Cognitive ageing, Development of the nervous system, Diseases of the nervous system, Neural ageing, Drug development, Neurological disorders, Dementia, Motor neuron disease, Neurodegeneration, Neurodegenerative diseases, Parkinson's disease, Cell biology, Drug discovery, Molecular biology, Neuroscience, Systems biology, Diseases, Pathogenesis, Mathematics and computing

## Abstract

Despite remarkable advances, research into neurodegeneration and Alzheimer Disease (AD) has nonetheless been dominated by inconsistent and conflicting theory. Basic questions regarding how and why the brain changes over time remain unanswered. In this work, we lay novel foundations for a consistent, integrated view of the aging brain. We develop neural economics—the study of the brain’s infrastructure, brain capital. Using mathematical modeling, we create ABC (*A*ging *B*rain *C*apital), a simple linear simultaneous-equation model that unites aspects of neuroscience, economics, and thermodynamics to explain the rise and fall of brain capital, and thus function, over the human lifespan. Solving and simulating this model, we show that in each of us, the resource budget constraints of our finite brains cause brain capital to reach an upper limit. The thermodynamics of our working brains cause persistent pathologies to inevitably accumulate. With time, the brain becomes damaged causing brain capital to depreciate and decline. Using derivative models, we suggest that this endogenous aging process underpins the pathogenesis and spectrum of neurodegenerative disease. We develop amyloid–tau interaction theory, a paradigm that bridges the unnecessary conflict between amyloid- and tau-centered hypotheses of AD. Finally, we discuss profound implications for therapeutic strategy and development.

## Introduction

Neurodegenerative diseases are chronic, progressive processes that present almost exclusively in late life^1–7^. They are in common characterized by the accumulation of various forms of protein aggregates accompanied by neurodegeneration. These neuropathological hallmarks herald pathogenic cascades^8–13^ from the molecular^14–16^ to systems^17^ level encompassing proteostatic stress^18^, neuroinflammation^19^, synaptic loss^9, 20^, neuronal dysfunction^9, 11^, and network failure^17, 21^, with genetic studies^22^ demonstrating that accumulation of the aggregates themselves are indeed the primary etiological driver^8, 23^. These processes evolve to distribute across the brain in characteristic patterns^24, 25^, causing commensurate patterns of functional decline^26, 27^—Alzheimer Dementia (AD), Parkinson Disease (PD), and other major forms of cognitive impairment.

It has become clear that the presence of these pathologies is not atypical but rather commonplace across the population^28–32^, dramatically differing in severity between individuals. Genotypes that predispose to a specific protein’s aggregation cause markedly accelerated deposition of that pathology^22^, still on the time-course of decades^23^. In the absence of genetic predisposition, severe pathology occurs in mid-life to late-life only^28, 29^, possibly without exception. Increasingly comprehensive research demonstrates that elderly brains are often severely marked by multiple forms of pathology^28, 33^. The result is a complex spectrum of neuropathology, and commensurately of neurological function, across the aging population^7, 34–37^.

Unfortunately, despite tremendous isolated mechanistic advances, these collective realities are not consistently integrated into understanding of disease pathogenesis within the neuroscientific community. What has resulted is a deep lack of unity^38^ with inconsistent concepts driving investigations, interpretation of data, and therapeutic development. Key questions regarding this spectrum of decline with brain aging and neurodegeneration remain unanswered. Why do these processes occur; how are they related; are they endogenous or environmentally driven; preventable or inevitable; how can they be treated, slowed, reversed. The lack of consensus on these paramount issues has doubtless contributed to the continued dearth of therapeutic success. The burden of neurodegenerative disease remains unmitigated.

Herein, we take a completely novel approach to answer these questions by developing neural economics, the study of the brain’s infrastructure, brain capital. We create a conceptual and mathematical framework that begins to explain and convincingly unify the processes of brain aging, neurodegenerative disease, and AD. We hope this framework facilitates shared understanding within the neuroscientific community.

## The scope and language of neural economics

At any point in time, a single brain comprises hundreds of billions of neurons arranged in a dense matrix of interconnectivity with even greater numbers of supporting cells shaping each neuron’s microenvironment. This matrix is the infrastructure that creates electromagnetic patterns that comprise the mind—our perception, heuristics, memories, decisions, and actions. Collectively, this object is dynamic, continuously adapting to and processing the environment on the level of milliseconds; plastic, physically developing and maintaining itself; and stable, preserving learning over the scale of a lifetime. Aging impacts the brain on each of these levels, with functional decline by no means a direct consequence of molecular or cellular phenomena^26^. To model such a process requires simplification yet preservation of its essential aspects. Reductionist molecular models of aging woefully fail to address neurological function^17^; conversely, approaches at the level of function without backing in underlying physiology are likewise incomplete. Profound advances in neuroscience are left nonetheless uninterpretable and poorly actionable in the absence of unifying context.

In response, we defined and developed the concept of neural economics (Box 1). Neural economics is the study of brain capital, the brain’s infrastructure, and provides a framework for the complexities of the brain’s development, organization, and dynamics. Brain capital is the organization of neuronal interconnectivity. The deployment of brain capital produces the mind that we each experience, live, and are. A simple, representative example of this interplay is in memory formation. In response to the environment, hippocampal neurons organize their connectivity into meaningful networks, brain capital. The deployment of these networks in response to associated cues generates the memory itself. Neural economics offers a uniquely rich lens on neuroscience, linking biology to function through the infrastructure that bridges them. As we describe herein, applying neural economics on the macro-level provides an explanation of brain aging, unifying pathogenesis to provide a unique lens on neurodegenerative disease as well as promising directions for therapeutic intervention.

Box 1: What is neural economics? Neural economics is the study of brain capital. Brain capital is the brain’s infrastructure, the organization of neuronal interconnectivity. At a basic level, brain capital is the bridge between mind and brain. Brain capital forms and refines through processes of neurodevelopment and neuroplasticity. The deployment of brain capital (i.e., the functioning of meaningful neuronal networks) produces our perception and behavior—the mind.

## The ABC model and the determinants of brain capital

In this study’s approach, we considered the brain as a finite physical asset that generates a stream of finite resources (endowment, E_t_). The brain’s endowment can be used as investment (I_t_) to generate and maintain brain capital (K_t_) which depreciates over time. Modeled recursively, this relationship is as follows:

1 $${\text{K}}_{{\text{t}}} = {\upalpha} \times {\text{K}}_{{{\text{t}} - 1}} + {\text{I}}_{{\text{t}}}$$

where α is a parameter associated with capital depreciation and K_0_ represents starting brain capital stock at the beginning of life. The above represents the fundamental process that determines the state of the levels of brain infrastructure at any point over a simulated lifespan.

In many ways, the active deployment of brain capital to generate neurological function is akin to a production process—the orchestrated activity of dynamic neuronal networks produces human experience. Any production process generates unwanted byproducts such as pollution and heat by the second law of thermodynamics. Likewise, we reasoned that the deployment of human brain capital must generate unwanted byproducts within the brain, collectively defined as pathology (P_t_). This concept is extensively based in neurobiology, with reactive oxygen species (ROS), misfolded proteins, and waste products all well-characterized byproducts of cellular operations^18, 32, 39^. In the brain, the efficient removal and repurposing of these unwanted byproducts is a must because unlike common real-world systems, many cannot be easily externalized and would otherwise rapidly accumulate in limited space causing significant damage^40^. We modeled this necessity as the brain redirecting available resources (a portion of endowment) as pathology control (PC_t_), e.g. the unfolded protein response. Importantly, however, the second law of thermodynamics again dictates that pollution control processes cannot be fully efficient, a well-known reality in environmental engineering. Indeed, certain forms of brain pathology are known to resist clearance mechanisms and persist^8, 39^. We modeled this essential consideration by representing pathology in two forms—short-term pathology (P^ST^_t_) that the brain is capable of clearing and long-term pathology (P^LT^_t_) that intrinsically persists. Cellular correlates of these pathologies include the distinction^18, 39, 41^ between misfolded proteins, short-term pathology that the cell compensates for and clears, and resilient protein aggregates, pathology that endures for decades^28^. Both forms of pathology are cumulative and produced proportionately to brain capital deployed. Modeled recursively:

2 $${\text{P}}_{{\text{t}}}^{{{\text{ST}}}} = {\text{(P}}_{{{\text{t}} - {1}}}^{{{\text{ST}}}} + {\upgamma}_{{{\text{ST}}}} \times {\text{K}}_{{\text{t}}} ) - {\text{PC}}_{{\text{t}}}$$

3 $${\text{P}}_{{\text{t}}}^{{{\text{LT}}}} = {\text{P}}_{{{\text{t}} - {1}}}^{{{\text{LT}}}} + {\upgamma}_{{{\text{LT}}}} \times {\text{K}}_{{\text{t}}}$$

where the γ’s are parameters representing the rate of production of each type of pathology. We then sought to capture the impact of pathology accumulation. Pathologies are directly detrimental to the brain via a plethora of mechanisms^14–16^. The decline in the brain asset due to pathology burden would be reflected in a decline in the resources generated by that asset each period:

4 $${\text{E}}_{{\text{t}}} = {\text{E}}_{{0}} {-}{\upbeta} \times {\text{(P}}_{{\text{t}}}^{{{\text{ST}}}} + {\text{P}}_{{\text{t}}}^{{{\text{LT}}}} {)}$$

where β is a parameter representing the brain’s vulnerability to pathology and E_0_ represents the endowment generated by the brain in its mint condition. The need for pollution control as well as the impact of accumulated pathology in turn determine the brain’s resource budget (endowment) available to either develop brain capital as investment or clear short-term pathology as pathology control. The brain’s resource budget is captured by the following:

5 $${\text{E}}_{{\text{t}}} = {\text{I}}_{{\text{t}}} + {\text{PC}}_{{\text{t}}}$$

**Figure 1:**
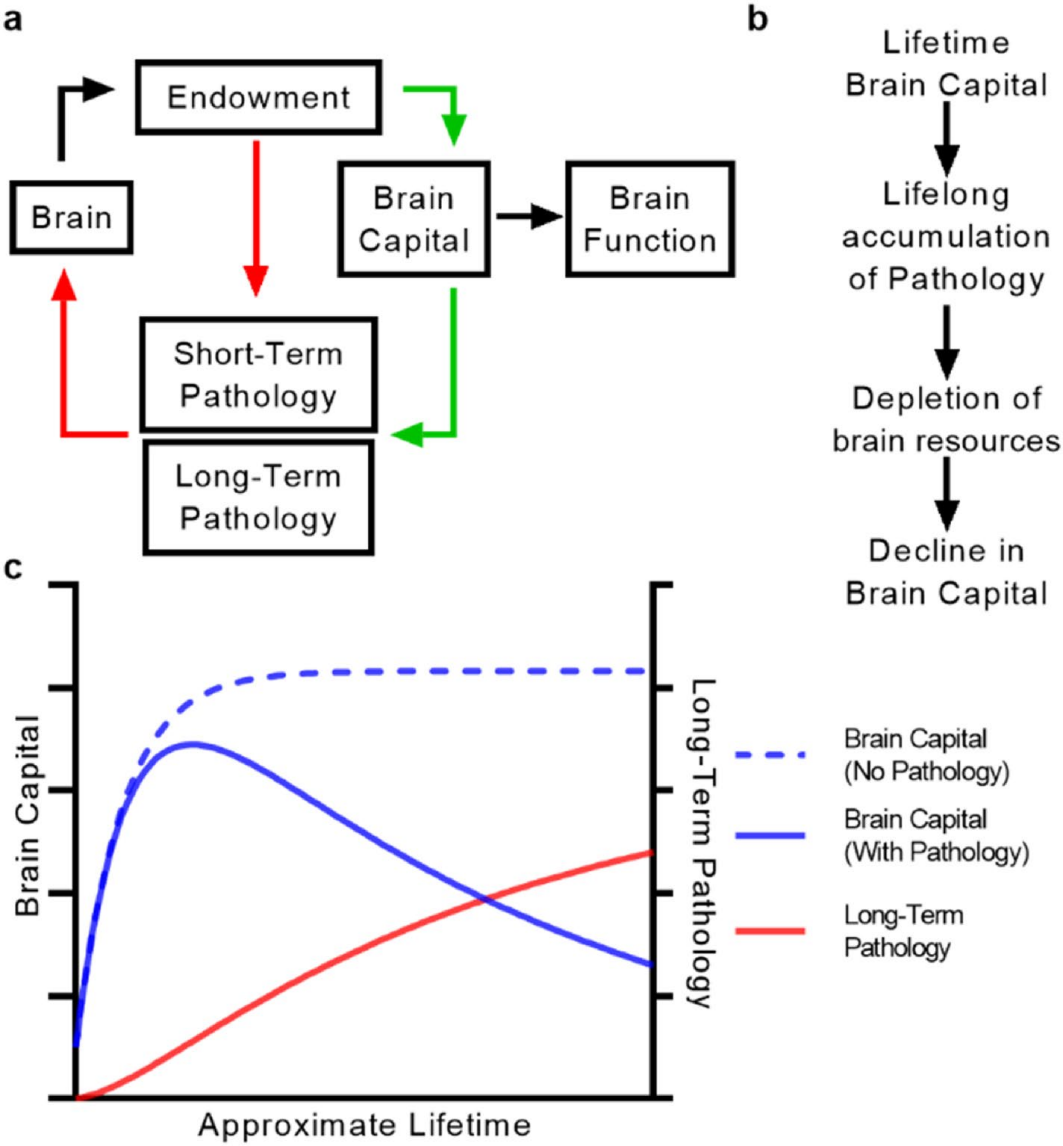
The ABC model defines a universal mechanism of brain aging. (**a**) Relationships between variables in ABC. Green arrows represent positive contributions (e.g. endowment increases brain capital over time via investment) whereas red arrows represent negative contributions (e.g. endowment clears short-term pathology via pathology control). (**b**) A simplified model for the basis of brain aging as emerges from ABC. (**c**) A representative simulation of brain capital and pathology evolution over an individual’s lifetime in ABC either without long-term pathology produced (dotted blue line) or with long-term pathology produced (solid blue line, pathology in red). All figure axes have arbitrary units.

Thus, the complete ABC (*A*ging *B*rain *C*apital) model (Box 2, Table 1) elegantly ties together essential co-evolving processes within the brain—the generation and maintenance of brain capital, the impact of its deployment on the production of pathology, the clearance and persistence of pathology, pathology-mediated damage, and the brain’s resource budget (Fig. 1A). It is crucial to recognize that the state variables of ABC are not intended to directly relate to observable phenomena or model the details of specific subprocesses. Instead, they reduce the uncharted complexities of neural structure and function into simple, quantifiable relationships that nonetheless cast deep insight into the processes they represent. Brain capital stock is not readily observable or measurable—yet as we demonstrate, its rise and fall within the ABC model is readily generalizable to the evolution of neurological function over the human lifespan.

**Table 1:** ABC variables and constraints.

Variable	Meaning	Constraints
K_t_	Brain capital	K_0_ > 0
E_t_	Brain endowment	E_0_ > 0
I_t_	Investment	N/A
PC_t_	Pathology control	N/A
P^ST^_t_	Short-term pathology	P^ST^_0_ = 0
P^LT^_t_	Long-term pathology	P^LT^_0_ = 0
α	Capital depreciation rate	< 1
β	Pathology impact	> 1
γ_ST_	Short-term pathology production rate	≥ 0
γ_LT_	Long-term pathology production rate	≥ 0

Box 2: The ABC model

- The brain is a finite physical asset that generates a finite stream of resources (endowment).
- Endowment can be budgeted as investment to generate brain capital.
- Brain capital depreciates over time.
- The deployment of brain capital generates byproducts (pathology) by the second law of thermodynamics.
- Endowment can be budgeted as pathology control.
- Pathology control cannot be completely efficient by the second law of thermodynamics.
- Thus, pathology takes two forms that are both produced in proportion to brain capital—short-term (clearable) and long-term (persistent) pathology.
- Pathology damages the brain, reflected in a decline in endowment proportional to pathology burden.
- Pathology control is prioritized over investment.

## The basis for and inevitability of brain aging

We obtained an analytical solution to the simultaneous-equation ABC system (“Methods”). We then simulated several foundational scenarios of human brain capital, defining key parameters to approximate the human lifespan. In the first scenario, we explored the development of brain capital in the complete absence of pathology (both values of γ set to zero). In this scenario, brain capital increases until it plateaus after a certain time period (Supplementary Fig. 1A). This is because the depreciation of capital ultimately limits its further development, with the brain’s resource budget being diverted from investment of new capital to maintenance of existing capital (Supplementary Fig. 1B). Thus, even in the absence of pathology, there is a limit to human brain capital.

We next considered the scenario where short-term but not long-term pathology is generated over time. Capital once again developed in early-life and plateaued (Supplementary Fig. 1C) with the need for pathology control additionally limiting the upper limit to brain capital (Supplementary Fig. 1C,D). Importantly, the brain in this state does not accumulate any pathology, as resources diverted to pathology control are consistently capable of matching and clearing any pathology generated.

We then simulated ABC in full by including the production of long-term pathology. The results of this scenario are remarkable for three major deviations from previous scenarios: firstly, long-term pathology accumulates over the lifespan (Fig. 1B,C, Supplementary Fig. 1E); secondly, endowment declines over the lifespan (Supplementary Fig. 1F), reflecting pathology-mediated depletion; lastly, although capital develops and plateaus as before, it declines in late life (Fig. 1B,C, Supplementary Fig. 1E). These results were remarkably robust to reasonable ranges for all model parameters. In this complete model, the relationships between the brain’s resource budget, capital development and depreciation, and pathology control are preserved. Additionally, long-term pathology, persistent due to the second law of thermodynamics, accumulates in an age-driven manner reflecting the development and deployment of brain capital. This accumulating pathology causes a proportional decline in endowment, representing depletion in the brain asset’s quality and resources (e.g. disrupted cellular operations^8, 12, 41^, neuroinflammation^19^, neuron loss, etc.). Decline in endowment crosses a crucial threshold where available investment is no longer sufficient to maintain depreciating levels of brain capital (Supplementary Fig. 1F) and consequently, capital in turn declines. This late-life decline in capital would manifest as brain network dysfunction and escalating functional loss in the affected systems. As the brain continues to decline, it generates fewer resources and thus progressively loses its ability to clear short-term pathologies, which may in turn accumulate (Supplementary Fig. 1G) and cause damage (Supplementary Fig. 1H).

Thus, ABC demonstrates that limits to brain capital, the accumulation of persistent pathologies, and brain capital decline with aging emerge as endogenous and inevitable consequences of thermodynamic necessity and the finite nature of the brain’s resources (Box 3). We suggest that these findings are generalizable to many characterized processes, from neuroplasticity^42^ to the accumulation of protein aggregates^8, 28, 30, 32^ to the loss of cognitive function in dementia. This model represents an integrated framework by which diverse mechanisms and subprocesses can be contextualized within the spectrum of brain aging across the population (Supplementary Fig. 2). Furthermore, as we present below, neurodegenerative disease can be thought of as subprocesses of aging within ABC, explaining their shared characteristics.

Box 3: Key implications of ABC

- The brain, and thus the brain’s capacity to develop, is finite—levels of brain capital must plateau.
- Persistent pathology inevitably accumulates over the brain’s lifespan by thermodynamic necessity.
- Due to persistent pathology, the quality of the brain as an asset progressively deteriorates over its lifespan.
- After hitting its peak, brain capital inevitably declines.
- Decline in brain capital with aging is endogenous.

## Neurodegeneration as part of brain aging: ABC-ND

The molecular basis for neurodegenerative disease centers around protein dyshomeostasis—the progressive accumulation of abnormally processed, dysfunctional proteins and their aggregation^8, 32^. This phenomenon, shared between seemingly disparate pathologies^14–16, 18^ and conditions^22, 24, 25^, is not unique to severe disease nor to the brain itself—rather, it is an essential hallmark of aging^32^. In the context of the brain, it is the etiological driver of multifactorial processes that underpin functional and cognitive decline.

We considered the pathogenesis of neurodegeneration through the lens of neural economics. The activity of human brain capital necessitates the continual functioning of its component parts. A central pillar of neuronal function is protein synthesis. Protein synthesis, however efficient, generates damaging byproducts due to thermodynamics. In response to this existential threat^40^, cellular quality control mechanisms adaptively engage^39, 41^ the vast majority of this short-term pathology. However, again thermodynamics dictates that this process cannot be completely efficient and does not completely clear protein pathology, the residue of which becomes compartmentalized as long-term pathology, persistent protein aggregates. Over time, these aggregates accumulate^8, 23, 28^, furthered by increasing inefficiencies in protein synthesis and quality control that occur by parallel mechanisms^18^. This process, including the accumulation of short-term misfolded proteins, progressively triggers the cellular phase of neurodegeneration^9–11, 13^ including the neuroinflammatory response^19^. In global context, these are endogenous, brain-wide processes^24, 25^, occurring at different rates and to different extents between pathologies, brain regions, and individuals due to only partially established genetic and environmental factors. In severe cases, involved brain regions, without capacity to regenerate progressive losses in neurons, become failing assets, no longer generating resources to maintain the infrastructure they underpin, which, unsupported, depreciates and collapses. The result, on the timescale of years, is commensurate functional decline. Decades of neurodegenerative disease research across disparate settings and levels becomes focused, contextualized, and integrated within the powerful schema of neural economics.

**Figure 2:**
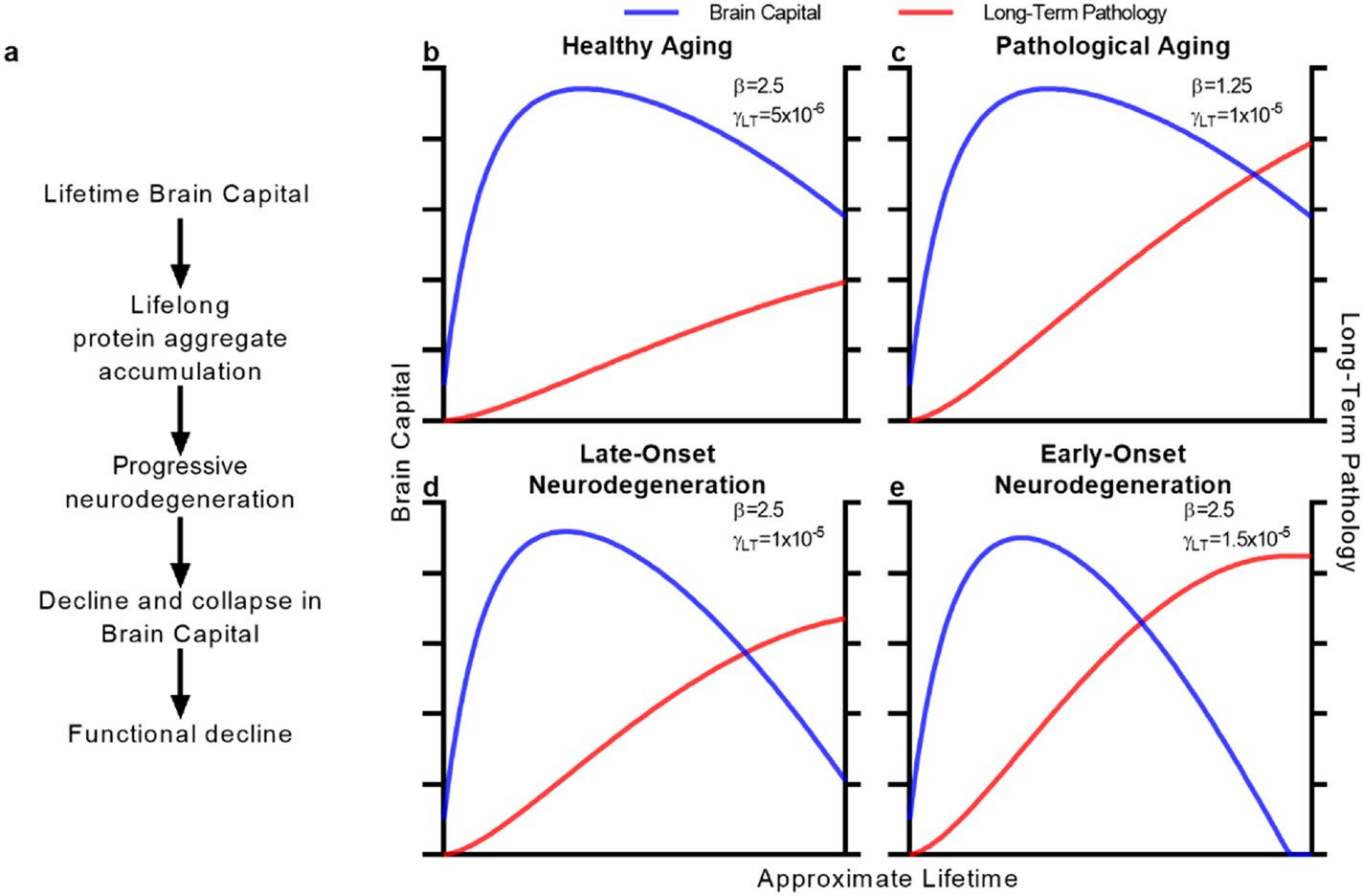
ABC-ND explains neurodegenerative disease as a subprocess of brain aging. (**a**) A basic framework for neurodegenerative aging, the subprocess by which lifetime deployment of brain capital causes the accumulation of specific protein aggregate pathologies and consequently, neurodegeneration. (**b**–**d**) Plausible simulations of the ABC-ND model that explain the spectrum of neurodegeneration including healthy aging (**b**), pathological aging (**c**), late-onset neurodegenerative disease (**d**), and familial disease (**e**). Parameters values varied between simulations are provided in the corresponding panels.

We straightforwardly modeled this general process of neurodegeneration due to a specific protein pathology (Fig. 2A) as a subprocess of aging, ABC-ND (ABC-*n*euro*d*egeneration, “Methods”, Box 4). The behavior of this model largely replicates the base ABC model (Supplementary Fig. 3A–F). Protein aggregate pathologies accumulate as long-term pathologies over the course of the lifetime (Supplementary Fig. 3E), causing progressive ‘neurodegeneration’, reflected in progressive decline in the brain’s available resources (Supplementary Fig. 3F).

A test of any model of neurodegeneration is its ability to explain the spectrum of pathology burden^7, 30, 34, 35, 43, 44^ and functional loss^36^ across the population. Aging and neurodegeneration are not linear processes—a certain age does not necessitate a certain pathology burden, a certain pathology burden a certain degree of neurodegeneration, even a certain degree of neurodegeneration corresponding functional decline. Largely unknown determinants shape individual trajectory.

Naturally, ABC-ND does not and could not incorporate these genetic, personal, environmental determinants of neurodegeneration. Rather, we found that it generates insights into how and why these contributors cause a single aging process to produce such an array of phenotypes. The defining parameters that underpin variable trajectory within ABC-ND are γ, the rate of production of long-term pathology, and β, the impact of that pathology. For instance, in an individual that has low rates of pathology accumulation and impact (γ and β, respectively), brain capital plateaus and remains relatively stable across the lifetime—a simulation of so-called ‘healthy aging’ (Fig. 2B) If that individual were to produce more pathology over their lifetime but remain relatively resistant to its impact, their brain capital would remain relatively preserved, as in individuals with ‘pathological aging’^37^ (Fig. 2C). In a simulated individual with high pathology burden in addition to sensitivity to its impact, brain capital significantly declines in late-life, the presentation of severe late-onset neurodegenerative disease (Fig. 2D). Finally, if genetic mutations cause artificially increased rates of pathology deposition, even with normal vulnerability to that pathology, a relatively early collapse in brain capital is observed, as in familial forms of neurodegeneration (Fig. 2E). Of course, these simplistic simulations are not intended to represent the actual mechanism of specific neurodegenerative diseases. But the striking conclusion of ABC-ND is that a single process of aging can plausibly reproduce and explain the spectrum of pathology, neurodegeneration, and functional decline across the population. Within this framework, so-called neurodegenerative disease is merely a severe manifestation.

Thus, we strongly suggest that neurodegenerative diseases do not represent distinct clinico-pathological entities from aging—rather, they form subprocesses of aging secondary to the production and impact of individual pathologies. Neurodegenerative disease should be more aptly considered a part of neurodegenerative aging. As such, it is an endogenous, inevitable process that ultimately stems from the brain’s finite resources and thermodynamic necessity. The presentation and severity of this process in each individual stems from largely unknown genetic and environmental determinants whose characterization and mechanism remain a crucial area of research for each disease.

Box 4: The ABC-ND model

- A model of neurodegeneration within ABC.
- The architecture of ABC encompassing the brain’s resource budget, the development of brain capital, and the production of pathology is preserved.
- Neurons are a non-renewable resource.
- Thus, decline in endowment due to pathology-mediated neurodegeneration is itself progressive.

## Alzheimer disease as part of brain aging: ABC-AD

Alzheimer disease (AD)^1^ is the leading neurodegenerative disease worldwide, yet an accepted disease mechanism remains lacking^23, 38, 43, 45^. Rather, dramatic controversies in theory are well-known even outside the field^38^. Part of the complexity of AD stems from the presence of two contributing pathologies^20^—senile plaques containing amyloid-β (Aβ) and neurofibrillary tangles containing abnormally processed tau. The classical amyloid hypothesis of AD was developed based on the observation that genetic mutations causing accelerated Aβ accumulation caused early-onset forms of AD, suggesting that the accumulation of Aβ pathology directly causes tau pathology, neurodegeneration, and functional decline in AD^45, 46^. This paradigm has nevertheless been criticized^31, 38, 47–49^ for failing to explain the significant accumulation of tau pathology in many individuals without or before Aβ deposits, so-called primary age-related tauopathy (PART)^31^. An additional complexity is that brain-wide patterns of Aβ, tau, and neurodegeneration differ^25, 47, 50, 51^, with tau consistently better correlated spatiotemporally with atrophy^47, 52^. This has led to prominent theories that claim that tau drives AD which nonetheless fail to explain the role of Aβ in familial AD and also the reality that mutations in tau causing accelerated tauopathy^15, 53^ are neither associated with Aβ nor AD but rather distinct patterns of neurodegeneration and functional decline. Many researchers cite various forms of synergy^54, 55^ between Aβ and tau to explain AD; others cite neuroinflammation^19^, neurovascular dysfunction^56, 57^, insulin resistance^58^, or heavy metals^59^ as the primary etiological drivers instead. This lack of unity in the field, despite decades of research and tremendous mechanistic advance, is perhaps the greatest failure in AD research. It has resulted in repeated therapeutic failures^60, 61^ and rapidly changing definitions of AD^43^.

We do not seek to attempt to synthesize or claim knowledge of the complete pathogenesis of AD. Indeed, integration cannot be achieved until troubling inconsistencies^38, 47^ in theory and mechanisms, generated across disparate disease models and settings, are clarified. Rather, we seek to establish and justify a framework for this pathogenesis that defines and explains AD across the entire population. By this means, the neuroscientific community can reapparaise its assumptions, disease models, and data, consistently integrate mechanisms, stabilize diagnostic criteria, and most importantly, develop promising therapies.

**Figure 3:**
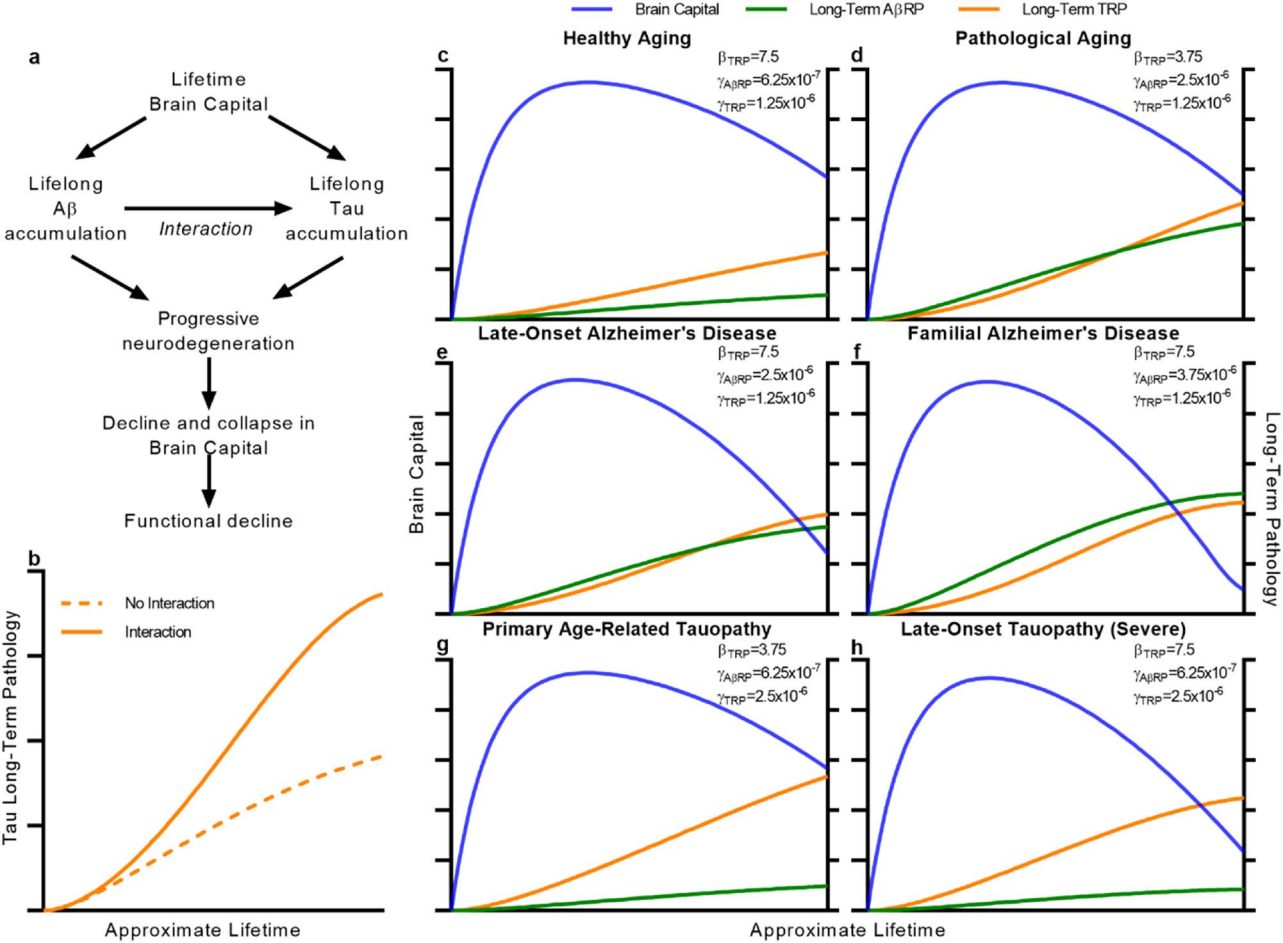
ABC-AD explains Alzheimer Disease as a subprocess of neurodegenerative aging. (**a**) A basic framework for amyloid–tau interaction theory and Alzheimer pathogenesis, the subprocess linking the lifetime deployment of brain capital, the accumulation of persistent forms of AβRP and TRP, neurodegeneration, and functional decline. (**b**) A representative simulation of the effect of amyloid–tau interaction on the accumulation of TRP comparing tau burden when the strength of interaction (δ) is zero (dotted line) vs. nonzero (solid line). (**c**–**h**) Plausible simulations of ABC-AD that explain the spectrum of Alzheimer disease including healthy aging (**c**), pathological aging (**d**), LOAD (**e**), fAD (**f**), primary age-related tauopathy (**g**), and late-onset tauopathy (**h**). Parameters values varied between simulations are shown in the corresponding panels. The value for δ was taken to equal to 1500 in all simulations.

We argue that much of the amyloid–tau debate is needless. The following truths^28, 46, 47^ are unambiguous. In the human population, Aβ pathology accumulates with aging with or without tau. Tau accumulates with or without Aβ. Mutations that accelerate Aβ accelerate tau accumulation^48, 62^. Mutations that accelerate tau do not accelerate Aβ. Mutations that accelerate Aβ accelerate patterns of functional decline classically defined as AD. Mutations that accelerate tau accelerate distinct patterns of functional decline. Neither the accumulation of Aβ, tau, or both forms of pathology is sufficient to cause AD-type functional decline. What logically, robustly follows from these facts is a relationship between amyloid and tau which we call amyloid–tau interaction. Simply, Aβ *accelerates* the accumulation of tau, a key distinction from the amyloid hypothesis. The beauty of amyloid–tau interaction as a core framework is its simplicity (Fig. 3A). As we demonstrate, it provides for the etiological roles of both Aβ and tau in the entire spectrum of AD pathogenesis, finally providing a means to reconcile amyloid- and tau-centered theories of AD.

Decades of research in flawed disease models have somewhat masked the reality that Aβ and tau are, in common, products of protein dyshomeostasis^14, 15, 46^. Though exact mechanisms differ^14, 15^ and are complicated by amyloid–tau interaction^45, 48, 54^, the laws of neural economics apply to independently explain the generation and impact of these pathologies as part of neurodegenerative aging. As we have described, thermodynamics dictates the generation and persistence of protein pathologies^63, 64^ which can accumulate to trigger neurodegeneration, the depreciation of unsupported brain capital, and ultimately, functional loss. To understand the impact of amyloid–tau interaction on this process, we created ABC-AD (ABC-*A*lzheimer *D*isease, “Methods”, Box 5, Table 2). ABC-AD is a two-pathology derivative of ABC-ND that simplistically models amyloid–tau interaction by stipulating that the burden of Aβ-related pathology (AβRP) causes a linear increase in the rate of accumulation of tau-related pathology (TRP) at any point in time. Again obtaining an exact analytic solution, we examined the behavior of system. In foundational simulations without either form of long-term pathology, brain capital evolved over the lifespan according to the base models ABC and ABC-ND (Supplementary Fig. 4A–D). In the presence of long-term TRP alone, ABC-AD reduced to the generalized single-pathology form of ABC-ND—TRP gradually accumulated driving a progressive decline in brain resources and capital (Supplementary Fig. 4E,F). In the reverse scenario (AβRP without TRP), the same results were produced (Supplementary Fig. 4G,H).

**Table 2:** ABC-AD variables and constraints.

Variable	Meaning	Constraints
K_t_	Brain capital	K_0_ > 0
E_t_	Brain endowment	E_0_ > 0
I_t_	Investment	N/A
${\text{PC}}_{{\text{t}}}^{{{\text{A}}{\upbeta} {\text{RP}}}}$	Amyloid pathology control	N/A
${\text{PC}}_{\text{t}}^{\text{TRP}}$	Tau pathology control	N/A
${\text{P}}_{{\text{t}}}^{{{\text{A}}{\upbeta} {\text{RPST}}}}$	Short-term amyloid pathology	${\text{P}}_{0}^{{{\text{A}}{\upbeta} {\text{RPST}}}}$ = 0
${\text{P}}_{{\text{t}}}^{{{\text{A}}{\upbeta} {\text{RPLT}}}}$	Long-term amyloid pathology	${\text{P}}_{0}^{{{\text{A}}{\upbeta} {\text{RPLT}}}}$ = 0
${\text{P}}_{\text{t}}^{\text{TRPST}}$	Short-term tau pathology	${\text{P}}_{0}^{\text{TRPST}}$ = 0
${\text{P}}_{\text{t}}^{\text{TRPLT}}$	Long-term tau pathology	${\text{P}}_{0}^{\text{TRPLT}}$ = 0
α	Capital depreciation rate	< 1
${\upbeta}_{{{\text{A}}{\upbeta} {\text{RP}}}}$	Amyloid pathology impact	≥ 0
${\upbeta}_{{{\text{TRP}}}}$	Tau pathology impact	≥ 0
${\upgamma}_{{{\text{ST}}}}^{{{\text{A}}{\upbeta} {\text{RP}}}}$	Short-term amyloid pathology production rate	≥ 0
${\upgamma}_{{{\text{LT}}}}^{{{\text{A}}{\upbeta} {\text{RP}}}}$	Long-term amyloid pathology production rate	≥ 0
${\upgamma}_{{{\text{ST}}}}^{{{\text{TRP}}}}$	Short-term tau pathology production rate	≥ 0
${\upgamma}_{{{\text{LT}}}}^{{{\text{TRP}}}}$	Long-term tau pathology production rate	≥ 0
δ	Amyloid–tau interaction strength	≥ 0

We then considered the complete scenario of ABC-AD with both AβRP and TRP present. In this case, AβRP and TRP gradually accumulate as a natural consequence of aging as expected. Because of amyloid–tau interaction, the growing presence of AβRP accelerates the production of TRP (Fig. 3B, Supplementary Fig. 5A,B). As in the base models, the accumulation of pathology drives decline in brain resources and consequently, a late-life depreciation of brain capital (Supplementary Fig. 6A,B). It is increasingly apparent that the brain may be more vulnerable to TRP than AβRP in AD^17, 28, 47^, potentially explaining disparate spatiotemporal associations of pathology burden with atrophy and neuron loss^47^. As can be modeled in ABC-AD, TRP has a greater total contribution to neurodegeneration, but Aβ has both a direct and indirect impact through amyloid–tau interaction (Supplementary Fig. 6B).

Explaining the complete spectrum of AD-related pathological change in the aging population has remained one of the greatest hurdles for contemporary hypotheses^23, 37, 38, 43, 44, 49^. As a testament to the validity of the amyloid–tau interaction theory, we found that this spectrum naturally emerges from ABC-AD. Healthy aging can be simulated as brain capital evolution with low production of both forms of pathology, resulting in fairly stable brain capital levels persisting through old age (Fig. 3C). Increased rates of accumulation of AβRP contribute to neurodegeneration and late-life decline in brain capital both directly and indirectly through acceleration of TRP accumulation. In a brain that is relatively resistant to TRP, this manifests as ‘pathological aging’^37^, with both pathologies accumulating in the relative absence of neurodegeneration (Fig. 3D). Otherwise, the accumulation of both pathologies causes severe late-life decline, a conceivable scenario for late-onset AD (LOAD) (Fig. 3E). In patients with familial AD (fAD) mutations that promote AβRP, the substantial increase in the rate of production of AβRP drives both an acceleration in TRP and a severe, early-onset decline in capital (Fig. 3F) as is observed. Importantly, these findings are entirely consistent with TRP-mediated neurodegeneration in the absence of AβRP. At a basic level, increasing TRP accumulation independently of Aβ may conceivably drive the range of functional decline observed from PART (Fig. 3G) to late-onset tauopathy (Fig. 3H). Realistically, several patterns of tauopathy exist that cause differing patterns of neurodegenerative disease^15, 31, 53^. It seems likely that the Braak stages of tau in AD^50^ reflect a specific mechanism and evolution of tau perhaps selectively promoted by amyloid–tau interaction^48^.

Again, these simplistic simulations are not intended as direct models of AD pathology or pathogenesis^9, 10, 23, 65^. Yet we suggest several key insights from these findings. To begin with, the accumulation and impact of both Aβ (Supplementary Figs. 6C,D, 7A) and tau (Supplementary Figs. 6E,F, 7B) can be thought as processes of neurodegenerative aging and thus, as subprocesses of brain aging. These two forms of pathology are linked at the very least by amyloid–tau interaction and severe AD is the product of their accumulation, interaction, and impact. Moreover, AD is not a distinct clinico-pathological entity^17, 34, 43, 49^. Rather, the accumulation of Aβ and tau and associated patterns of cognitive decline, in other words Alzheimer pathogenesis, exists as a spectrum across the population. This spectrum, a product of multifaceted determinants, can be conceived as differential manifestations of a single pathogeneic process, an inevitable, endogenous product of the brain’s finite resources and thermodynamic necessity. This conceptual framework does not draw clean lines between ‘diseased’ and ‘healthy’ individuals. Rather, it more realistically encompasses and explains Alzheimer Spectrum Disease as part of aging.

Box 5: The ABC-AD model

- A two-pathology derivative of ABC-ND.
- The architecture of ABC encompassing the brain’s resource budget, the development of brain capital, and the production of pathology is preserved.
- Amyloid–tau interaction theory is simplistically represented as acceleration in the rate of accumulation of tau in proportion to current amyloid burden.
- As in ABC-ND, decline in endowment due to pathology-mediated neurodegeneration is progressive.

## Therapeutic strategy and a call for unity

Understanding brain aging, neurodegeneration, and AD is not merely an academic pursuit. In the absence of existing therapeutics that are readily effective, therapeutic advance can only stem from purposeful design and development. Successful disease-modifying therapies stem from identification of so-called ‘druggable’ targets^66^, the pillars and vulnerabilities of pathogenesis. In a final investigation, we sought to illuminate such targets and in so doing, cast light on the repeated failure of attempted therapies to date.

Neural economics suggests that neurodegeneration is an exceptionally challenging process to attempt to treat. Unlike infection, it is not exogenous. Unlike inflammation, it is not intrinsically reversible. Unlike cancer, it is not an aberration. There is no target for antibiotics or antineoplastics, no pathologic immune activation responsive to corticosteroids, no receptors for autonomic modulation. Instead, the task is to repair an incredibly complex failing system, an asset stripped of its nonrenewable resources, lost or inexorably deteriorating infrastructure. Treating and curing neurodegenerative disease represents an immense challenge, a true gauntlet for modern science. The stakes are high—success in this endeavor would mean incalculable reductions in disability and recovery of cognition and function for millions.

**Figure 4:**
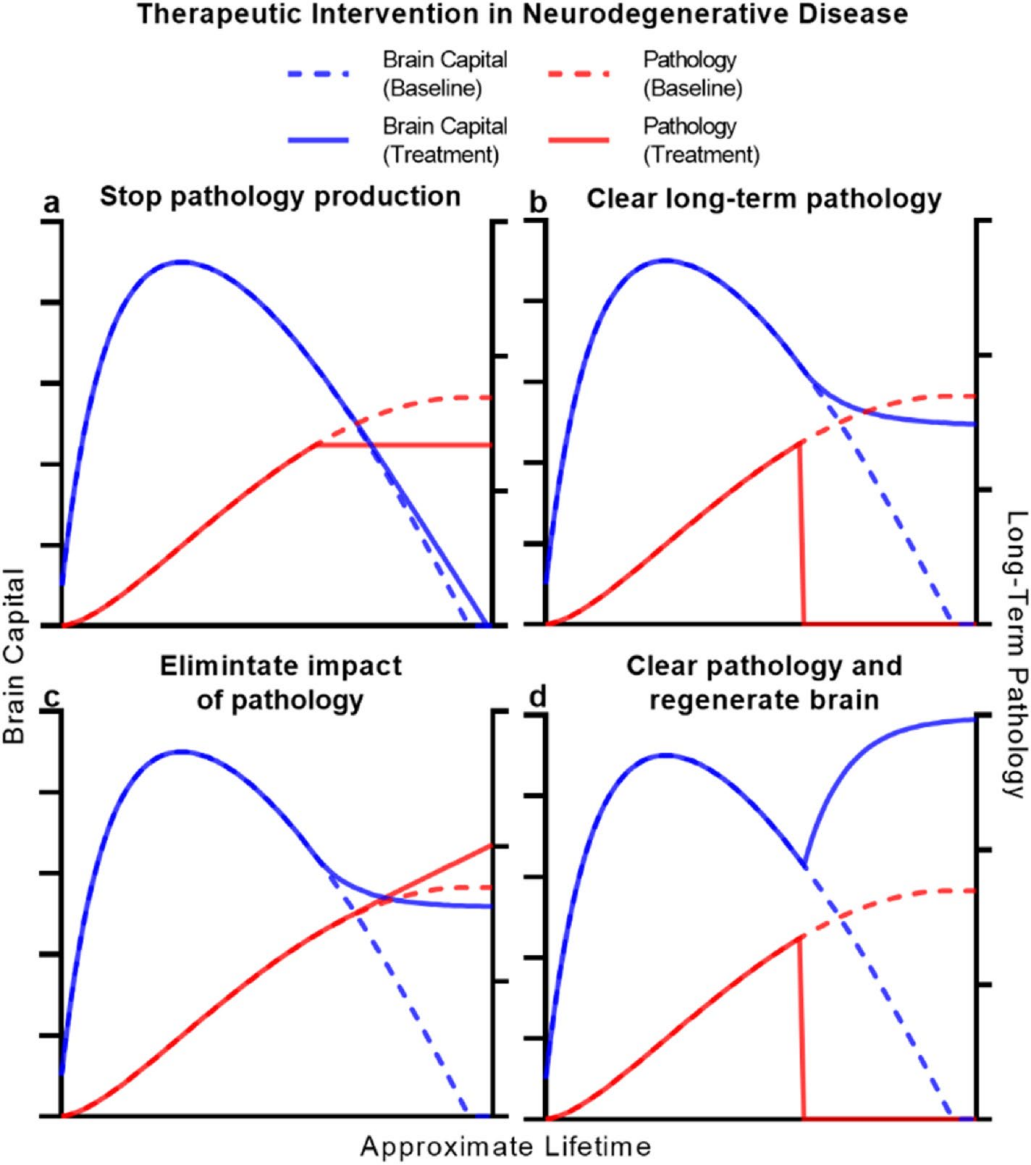
Simulating the role of disease-modifying therapy in neurodegeneration. (**a**–**d**) Simulations of ‘perfect’ therapeutic intervention in the ABC-ND model of neurodegeneration. The evolution of brain capital and pathology burden in a familial neurodegenerative disease scenario was simulated after stopping pathology production (**a**), permanently clearing all pathology (**b**), eliminating the impact of pathology (**c**), or both clearing pathology and permanently regenerating brain endowment (**d**). Each panel shows the evolution of brain capital (blue) and pathology (red) without (dotted line) and with (solid line) the target intervention.

We used simulations of ABC-ND to provide insight into how different therapeutic approaches might counter the progression of pathology and capital decline in a simulated patient with severe neurodegenerative disease. We represented therapy as an instantaneous modification to model parameters at a particular time period (“Methods”), an obvious but nonetheless revealing simplification of each approaches’ capabilities. We began by considering an approach that reduces or halts the production of pathology, something strived for by many in the neuroscientific community^45, 67, 68^. However, we found that even a therapeutic that completely eliminates any production of new short-term and long-term pathology marginally slows but fails to stop consequent decline in brain capital (Fig. 4A). This stems from the nature of pathology and its impact in ABC-ND. Even when production of pathology is stopped, pre-existing long-term pathology persists to cause damage. Furthermore, pre-existing neurodegeneration persists. What results is continued and largely unmitigated decline in the brain’s resources and thus, depreciation of brain capital. We believe that this important limitation in such a widely sought-after approach deserves greater consideration and recognition.

We then considered other therapeutic approaches within ABC-ND. We simulated a pathology-clearing therapeutic—one that physically clears the offending long-term pathology, removing or otherwise repurposing all of it by some novel mechanism^69, 70^. A perfect therapeutic with such properties is capable of halting further neurodegeneration and thus, further decline in capital (Fig. 4B), the brain eventually reaching a steady-state where its depleted but stable resources maintain brain capital. The same result is reached by a theoretical therapeutic that completely blocks the impact of pathology on the brain, eliminating the brain’s vulnerability. In this scenario, brain capital stabilizes despite the growing presence of pathology (Fig. 4C). Realistically, however, multiple overlapping mechanisms will cause the presence of pathology to cause dysfunction and damage. This is a fatal flaw of selective neuroprotective strategies^71^—they do not modify the underlying disease process and are thus ineffective to dampen the wave of disease impact, possibly explaining the repeated failure of these approaches.

**Figure 5:**
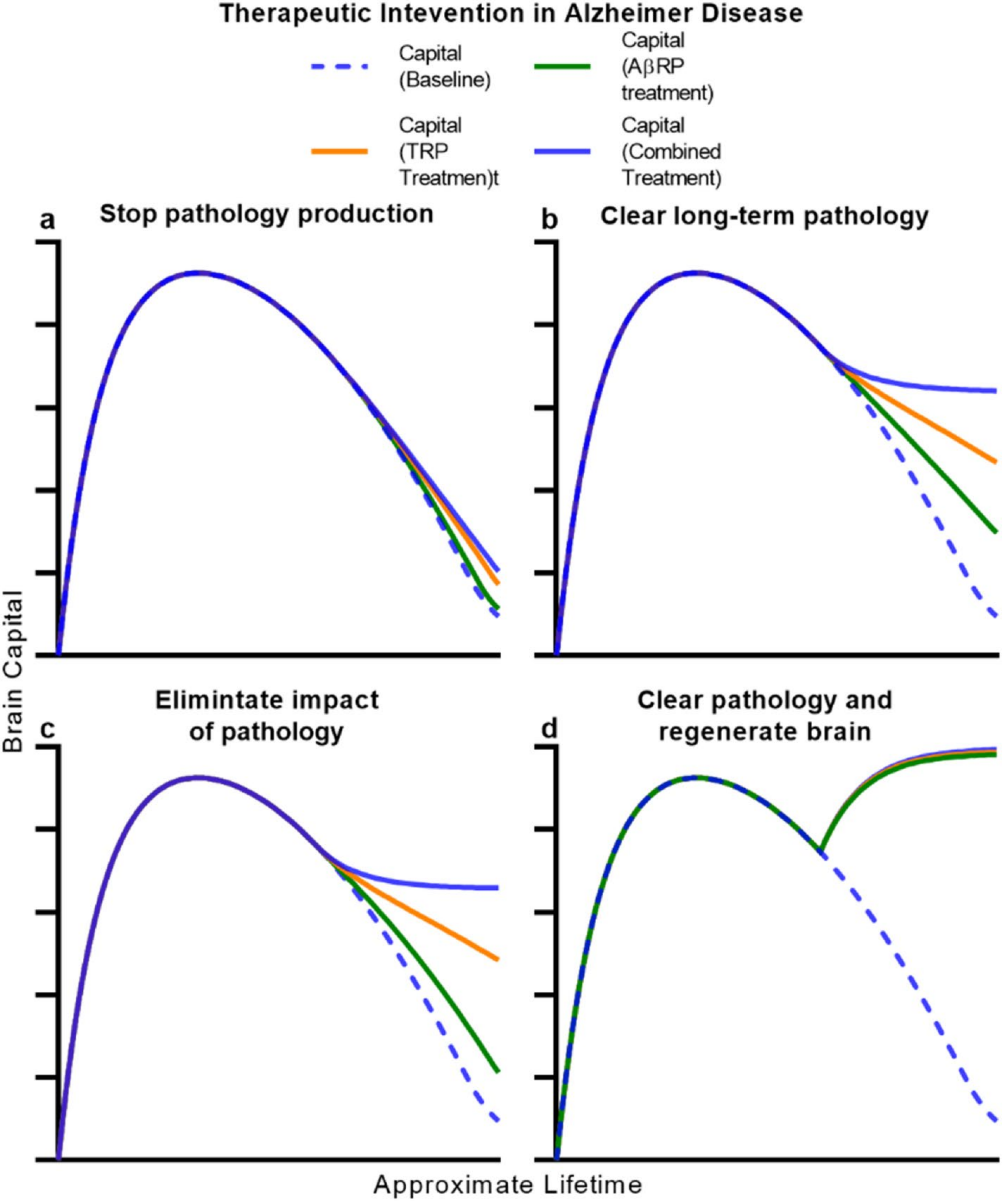
Simulating the role of disease-modifying therapy in Alzheimer Disease. (**a**–**d**) Simulations of ‘perfect’ therapeutic intervention in the ABC-AD model of Alzheimer Disease. The evolution of brain capital and pathology burden in a familial Alzheimer Disease scenario was simulated after stopping pathology production (**a**), permanently clearing pathology (**b**), eliminating the impact of pathology (**c**), or both clearing pathology and permanently regenerating brain endowment (**d**). Each panel shows the evolution of brain capital at baseline (dotted blue line) and when the intervention is directed at amyloid (solid green line), tau (solid orange line), or both (solid blue line) pathologies.

We applied these same principles in ABC-AD to understand how therapeutic approaches can be predicted to shape functional decline in AD. Again, we found that stopping the production of AβRP, TRP, or both pathologies together is not sufficient to prevent decline (Fig. 5A). Results for pathology-clearing therapeutics in this model were more nuanced and dependent on the brain’s relative vulnerability to each pathology. Modeling tau as the major contributor to neurodegeneration, as extensive precedent suggests^47, 48^, we found that even complete clearance of AβRP is not sufficient to prevent capital decline (Fig. 5B)—in the absence of Aβ, TRP continues to accumulate and cause damage, although the rate and extent of decline was reduced. This is because amyloid–tau interaction suggests that the pathogenic process of tau-mediated neurodegeneration is merely accelerated by Aβ. TRP-clearing therapeutics^72^ would be expected to more significantly slow neurodegeneration and functional decline assuming the brain’s vulnerability to TRP is higher (Fig. 5B), although we found that only combination-clearance therapy of both pathologies halts AD-related decline entirely (Fig. 5B). Amyloid–tau interaction theory suggests that Alzheimer Spectrum Disease is driven by the combination of AβRP and TRP—as such, we propose that therapeutics designed to target only a single pathology cannot be expected to fully address decline, regardless of the brain’s relative vulnerability to each. These general results were replicated for impact-targeting approaches as well (Fig. 5C). Importantly, these findings explain much of the basis for the failure of anti:Aβ therapeutics^60, 61, 70^ and their marginal impact on the progression of AD.

We observed that even complete clearance of pathology in ABC-ND or combination-clearance in ABC-AD, goals certainly not within immediate reach, were not sufficient to reverse lost brain capital (and hence neurological function). This reflects a key facet of neurodegenerative disease, the reality that neurons are effectively nonrenewable resources. Their loss entails a permanent reduction in the brain’s ability to support levels of brain capital. Once neurodegeneration occurs, the brain lacks resources to rebuild capital even with a pathology-clearing therapeutic on board. Rather, these approaches are best suited to prevent disease progression and functional decline at an early stage (Supplementary Fig. 8). In both ABC-ND and ABC-AD, only replenishing brain endowment—that is, repairing the underlying asset and regenerating the brain’s resources—can reverse severe decline in capital by allowing the brain to rebuild its infrastructure (Figs. 4D, 5D). Due to the progressive nature of neurodegenerative diseases, their only cure lies in regenerative medicine^73, 74^ (Supplementary Fig. 8). We believe development and promulgation of such a cure can only stem from the integration of stem cell biology, neurobiology, neuroimmunology, neurophysiology, tissue engineering, systems neuroscience, and neurosurgery and thus the unified, interdisciplinary efforts of the neuroscientific community. Effective and equitable care for the increasing population of patients suffering from the functional consequences of brain aging, regardless of available treatments, requires every element of the interdisciplinary clinical care team^27^. On all fronts, the global burden of neurodegenerative disease calls for unprecedented collaboration and unity.

Through this work, we hope to establish a framework by which the disparate branches of neuroscientific research can be reappraised and integrated. Of course, there are key limitations to our investigations and findings. Neural economics is a schema and brain capital is not observable or readily quantifiable. The ABC models are models of processes, not the processes themselves. In modeling neurodegenerative disease and AD we do not attempt to approximate clinicopathological data although the trajectories we simulate can be readily generalized to many findings. The concepts of pathogenesis we present are merely backbones by which rich mechanisms of pathobiology can be contextualized. We believe the power of neural economics is that its cuts to the foundational concepts and relationships that define the brain and thus, how and why the brain changes over the lifespan. In future, our work can be applied to guide efforts at neuroregeneration or be further developed to cast insight into the basic structure of the human mind.

## Methods

### Solutions to ABC, ABC-ND, and ABC-AD behavior

The behavior of the ABC model is determined by the brain’s resource budget-constraint (Eq. 5). In the complete absence of pathology, the brain’s endowment devotes entirely to investment and is constant over time. In the presence of short-term pathology, the brain must ‘choose’ its prioritization of investment and pathology control. We specified two assumptions in order to arrive at model behavior. Firstly, we assumed that the brain will distribute its endowment to maximize brain capital. Secondly, we made an assumption about the impact of pathology on the brain. We believe that the presence of pathology costs the brain more resources than those required to clear that pathology—in other words, it pays for the brain to clear pathology. Mathematically, we specified this by constraining the parameter β to be greater than one across all models. Even if this second assumption does not hold true, the model’s behavior is preserved. Three conceivable cases may emerge over an individual simulation—firstly, a case where the brain is capable of clearing all short-term pathology; secondly, where the brain can only partially clear pathology; and lastly, where the brain has no resources to clear short-term pathology.

#### Case 1

In the first case, sufficient resources are devoted to pathology control and the remainder to investment, such that:

6 $${\text{I}}_{{\text{t}}} > {0}$$

7 $${\text{PC}}_{{\text{t}}} = {\upgamma}_{{{\text{ST}}}} \times {\text{K}}_{{\text{t}}} + {\text{P}}_{{{\text{t}} - {1}}}^{{{\text{ST}}}}$$

8 $${\text{P}}_{{\text{t}}}^{{{\text{ST}}}} = {0}$$

A recursive solution to the evolution of capital over time can be obtained:

9 $${\text{K}}_{{\text{t}}} = \frac{{{\upalpha} \times {\text{K}}_{{{\text{t}} - {1}}} + {\text{E}}_{{0}} - {\upbeta} \times {\text{P}}_{{{\text{t}} - {1}}}^{{{\text{LT}}}} }}{{{1} + {\upgamma}_{{{\text{ST}}}} + {\upbeta} \times {\upgamma}_{{{\text{LT}}}} }}$$

Long-term pathologies, endowment, investment, and pathology control can all be solved for simultaneously given Eqs. (6–9).

#### Case 2

In the second case, all available endowment is directed to pathology control. Investment is therefore zero:

10 $${\text{I}}_{{\text{t}}} = {0}$$

11 $${\text{PC}}_{{\text{t}}} = {\text{E}}_{{\text{t}}}$$

12 $${\text{P}}_{{\text{t}}}^{{{\text{ST}}}} = {\upgamma}_{{{\text{ST}}}} \times {\text{K}}_{{\text{t}}} + {\text{P}}_{{{\text{t}} - {1}}}^{{{\text{ST}}}} - {\text{E}}_{{\text{t}}}$$

In this case, the evolution of capital over time is simply determined by capital depreciation:

13 $${\text{K}}_{{\text{t}}} = {\upalpha} \times {\text{K}}_{{{\text{t}} - {1}}}$$

Again, the co-evolution of other variables in the system can be analytically solved based on Eqs. (10–13).

#### Case 3

In the last case, the brain has no resources to clear pathology, and the brain’s endowment reduces below zero:

14 $${\text{I}}_{{\text{t}}} < {0}$$

15 $${\text{PC}}_{{\text{t}}} = {0}$$

16 $${\text{P}}_{{\text{t}}}^{{{\text{ST}}}} = {\upgamma}_{{{\text{ST}}}} \times {\text{K}}_{{\text{t}}} + {\text{P}}_{{{\text{t}} - {1}}}^{{{\text{ST}}}}$$

We interpreted a negative endowment as representing a fundamental crisis in the brain—the underlying asset that supports capital is collapsing, and so effectively the contribution from the brain itself is negative. As such, capital takes the following solution:

17 $${\text{K}}_{{\text{t}}} = \frac{{{\upalpha } \times {\text{K}}_{{{\text{t}} - {1}}} + {\text{E}}_{{0}} - {\upbeta} \times {\text{(P}}_{{{\text{t}} - {1}}}^{{{\text{LT}}}} + {\text{P}}_{{{\text{t}} - {1}}}^{{{\text{ST}}}} {)}}}{{{1} + {\upbeta} \times \left( {{\upgamma}_{{{\text{ST}}}} + {\upgamma}_{{{\text{LT}}}} } \right)}}$$

Given Eq. (17), the continued decline in endowment and accumulation of pathologies can be solved for to yield the complete behavior of the system. In each simulation of ABC and subsequent models, the system’s behavior in terms of the above scenarios was simultaneously determined at a given time point. The system’s evolution through each case naturally emerged and was not arbitrarily determined.

Neurodegeneration represents a unique case of damage to brain asset quality because it is in itself cumulative. In ABC, long-term pathologies are effectively equivalent to the damage they cause to the brain. Applying ABC specifically to the subprocess of neurodegenerative aging, the long-term pathologies (protein aggregates) and the damage they cause (neurodegeneration) are distinct. Because the brain cannot seem to reverse neuronal loss, it is more realistic to assume that neurodegeneration-causing protein aggregates cause damage in a given period that is proportional to total pathology load and that this damage in itself accumulates. The sole difference between the generalized framework of aging in ABC and the derivative model ABC-ND is specifically how the impact of pathology is modeled:

18 $${\text{E}}_{{\text{t}}} = {\text{E}}_{{{\text{t}} - {1}}} {-}{\upbeta} \times {\text{(P}}_{{\text{t}}}^{{{\text{ST}}}} + {\text{P}}_{{\text{t}}}^{{{\text{LT}}}} {)}$$

compared to the form of endowment in the ABC model (Eq. 4). The budget-constraint aspect of ABC-ND is identical to the original model, and the dynamic behavior of the solution is again determined by the value of endowment relative to total produced short-term pathology. The equivalent forms of Eqs. (9), (13), and (17) can be solved to obtain Eqs. (19–21), respectively, and yield highly comparable behavior:

19 $${\text{Case}}\;1{:}\quad {\text{K}}_{{\text{t}}} = \frac{{{\upalpha} \times {\text{K}}_{{{\text{t}} - {1}}} + {\text{E}}_{{{\text{t}} - {1}}} - {\upbeta} \times {\text{P}}_{{{\text{t}} - {1}}}^{{{\text{LT}}}} }}{{{1} + {\upgamma}_{{{\text{ST}}}} + {\upbeta} \times {\upgamma}_{{{\text{LT}}}} }}$$

20 $${\text{Case}}\;2{:}\quad {\text{K}}_{{\text{t}}} = {\upalpha} \times {\text{K}}_{{{\text{t}} - {1}}}$$

21 $${\text{Case}}\;3{:}\quad {\text{K}}_{{\text{t }}} = \frac{{{\upalpha} \times {\text{K}}_{{{\text{t}} - {1}}} + {\text{E}}_{{{\text{t}} - {1}}} - {\upbeta} \times {\text{(P}}_{{{\text{t}} - {1}}}^{{{\text{LT}}}} + {\text{P}}_{{{\text{t}} - {1}}}^{{{\text{ST}}}} {)}}}{{{1} + {\upbeta} \times \left( {{\upgamma}_{{{\text{ST}}}} + {\upgamma}_{{{\text{LT}}}} } \right)}}$$

ABC-AD expands upon ABC-ND in two major points: firstly, pathology is split into two components (AβRP and TRP) with each having short-term and long-term forms; secondly, amyloid–tau interaction is assumed (Fig. 3A). The exact quantitative relationship between AβRP and TRP is not currently known and is likely complex. We assumed that amyloid–tau interaction manifests as acceleration in the rate of long-term TRP directly proportional to AβRP burden up to the previous time-period:

22 $${\upgamma}_{{\text{t}}}^{{{\text{TRPLT}}}} = {\upgamma}_{{0}}^{{{\text{TRPLT}}}} \times \left( {{1} + {\updelta} \times {\text{A}}{\upbeta} {\text{RP}}_{{{\text{t}} - {1}}}^{{{\text{LT}}}} } \right)$$

where δ represents the degree of amyloid–tau interaction. The resource-budget constraint is slightly more complex in ABC-AD compared to the base models. Endowment distributes between investment, AβRP control, and TRP control. In the scenario where endowment is greater than the sum of total produced short-term pathologies, the solution for capital evolution over time is akin to Eqs. (9) and (19) (ABC and ABC-ND, respectively):

23 $${\text{K}}_{{\text{t}}} = \frac{{{\upalpha} \times {\text{K}}_{{{\text{t}} - {1}}} + {\text{E}}_{{{\text{t}} - {1}}} - {\upbeta}_{{{\text{A}}{\upbeta} {\text{RP}}}} \times {\text{A}}{\upbeta} {\text{RP}}_{{{\text{t}} - {1}}}^{{{\text{LT}}}} - {\upbeta}_{{{\text{TRP}}}} \times {\text{TRP}}_{{{\text{t}} - {1}}}^{{{\text{LT}}}} }}{{{1} + {\upgamma}_{{{\text{ST}}}}^{{{\text{A}}{\upbeta} {\text{RP}}}} + {\upgamma}_{{{\text{ST}}}}^{{{\text{TRP}}}} + {\upbeta}_{{{\text{A}}{\upbeta} {\text{RP}}}} \times {\upgamma}_{{{\text{LT}}}}^{{{\text{A}}{\upbeta} {\text{RP}}}} + {\upbeta}_{{{\text{TRP}}}} \times {\upgamma}_{{\text{t}}}^{{{\text{TRPLT}}}} }}$$

When endowment is insufficient to clear both AβRP and TRP, the brain prioritizes clearance of the pathology to which it has higher vulnerability (β). For example, in the simulations where brain vulnerability to TRP is greatest, investment is zero, PC first distributes to short-term TRP, and the remainder to short-term AβRP. In the case that endowment is lower than short-term TRP and greater than zero, all endowment is distributed to TRP control, investment is zero, short-term TRP becomes the difference between produced and cleared, and short-term AβRP is produced without clearance. In each of these scenarios, capital evolution takes the form of Eqs. (13) and (20). Finally, in the case where endowment is negative, the evolution of capital over time takes the following form, akin to Eqs. (8) and (12) in ABC and ABC-ND, respectively:

24 $${\text{K}}_{{\text{t}}} = \frac{{{\upalpha} \times {\text{K}}_{{{\text{t}} - {1}}} + {\text{E}}_{{{\text{t}} - {1}}} - {\upbeta}_{{{\text{A}}{\upbeta} {\text{RP}}}} \times \left( {{\text{A}}{\upbeta} {\text{RP}}_{{{\text{t}} - {1}}}^{{{\text{LT}}}} + {\text{A}}{\upbeta} {\text{RP}}_{{{\text{t}} - {1}}}^{{{\text{ST}}}} } \right) - {\upbeta}_{{{\text{TRP}}}} \times \left( {{\text{TRP}}_{{{\text{t}} - {1}}}^{{{\text{LT}}}} + {\text{TRP}}_{{{\text{t}} - {1}}}^{{{\text{ST}}}} } \right)}}{{{1} + {\upbeta}_{{{\text{A}}{\upbeta} {\text{RP}}}} \times \left( {{\upgamma}_{{{\text{LT}}}}^{{{\text{A}}{\upbeta} {\text{RP}}}} + {\upgamma}_{{{\text{ST}}}}^{{{\text{A}}{\upbeta} {\text{RP}}}} } \right) + {\upbeta}_{{{\text{TRP}}}} \times \left( {{\upgamma}_{{\text{t}}}^{{{\text{TRPLT}}}} + {\upgamma}_{{{\text{ST}}}}^{{{\text{TRP}}}} } \right)}}$$

### Simulating ABC, ABC-ND, and ABC-AD

In all simulations, parameters are assumed to be constant (i.e. equivalent to their initial value) over the lifetime, ensuring that the behavior of the system is truly endogenous. Appropriate, reasonable values for initial parameters were estimated based on known information about the underlying process. Tables 1 and 2 provide the ‘hard’ constraints we applied on model parameters.

### Simulating therapeutic intervention in ABC-ND and ABC-AD

Real therapeutics have complex, uncertain, and imperfect impact on variables within a physiological system such as the brain. We sought to define the upper limits of achievable therapeutic possibilities by simulating the effects of ‘perfect’ therapeutics—that is, interventions that achieve 100% efficacy. Rather than predicting the effectiveness of specific therapeutics, we used the ABC models to delineate the maximal effectiveness of a certain therapeutic approach. We simulated a perfect therapeutic that aims to stop the production of pathology by reducing the γ parameter associated with that pathology to zero at a particular time-point and following the behavior of the simulation following this intervention. We simulated complete clearance of pathology by instantaneously reducing total pathology levels to zero, and simulated eliminating the brain’s vulnerability to pathology by reducing the β parameter to zero. We modeled regeneration of the brain asset as an instantaneous recovery of endowment back to E_0_, the brain’s endowment in its mint condition. We modeled these interventions in ABC-ND and ABC-AD in the context of simulations of familial neurodegenerative disease and fAD respectively (Figs. 2E, 3F).

## Supplementary Information

Supplementary Figures.

## Data Availability

Data sharing not applicable to this article as no datasets were generated or analyzed during the current study. Model solutions, parameter values, and simulations will all be made available upon reasonable request.
