# Supplementary material for: The neural economics of brain aging
Source: Sci Rep. 2021 Jun 9;11:12167. doi: 10.1038/s41598-021-91621-5 (PMC8190309; doi:10.1038/s41598-021-91621-5)
Supplement: Supplementary file 1 — Supplementary Figures. [file 41598_2021_91621_MOESM1_ESM.docx]

***Supplementary Information***

Jacob Kosyakovsky***

*University of Virginia School of Medicine, 200 Jeanette Lancaster Way, Charlottesville, VA 22903, USA*

**jk6bw@hscmail.mcc.virginia.edu*


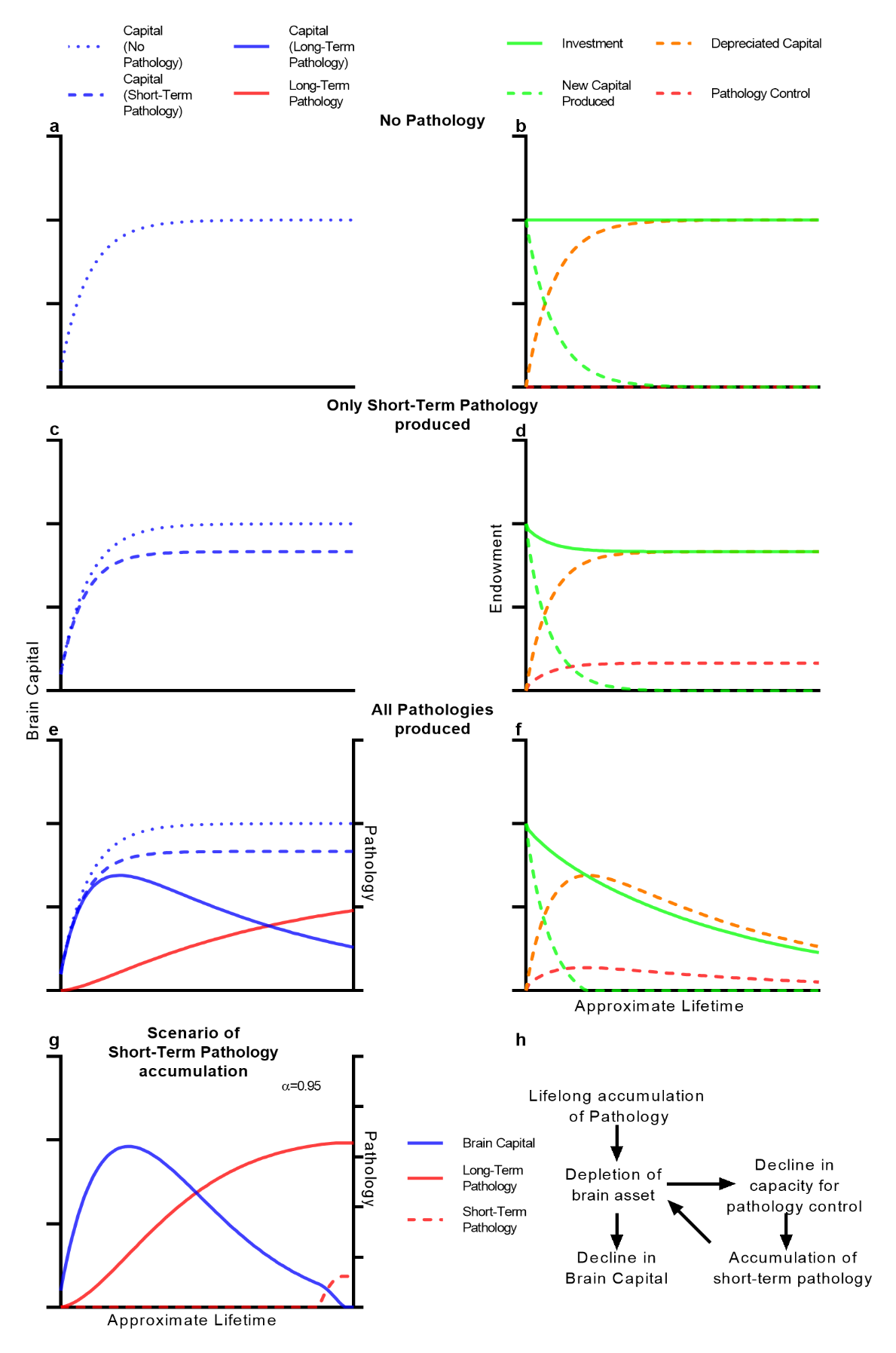


**Supplementary Figure 1. Behavior of the ABC model**

**Supplementary Figure 1. Behavior of the ABC model. a**, Representative simulation of the evolution of brain capital over the lifespan in the absence of either short-term or long-term pathology. **b**, Investment (solid green) gradually distributes from producing new brain capital (dotted green) to maintaining depreciated brain capital (dotted orange), driving the plateau in total brain capital. **c**, In the presence of short-term pathology, brain capital again plateaus, although at a lower level than the no-pathology scenario. **d**, Investment into new brain capital declines faster with the additional need for pathology control (dotted red). **e**, When long-term pathology (solid red) is produced, it accumulates over the lifespan, driving a decline in brain capital. **f**, Resources available for investment into brain capital ultimately decline and are outstripped by brain capital depreciation. This difference is the basis for loss of brain capital with aging. **g**, In simulations assuming a lesser degree of brain capital depreciation, short-term pathology accumulates at the end of life (described in **h**).

**Supplementary Figure 2. A simplified model of the spectrum of brain aging**

**Supplementary Figure 2. A simplified model of the spectrum of brain aging.** The set of aging-related long-term pathologies accumulates in every individual and brain region at different rates. Conceivably, the observed clinico-pathologic spectrum of brain aging could emerge from this variability. The determinants of this spectrum are genetic, intrinsic, and environmental factors specific to each pathology.


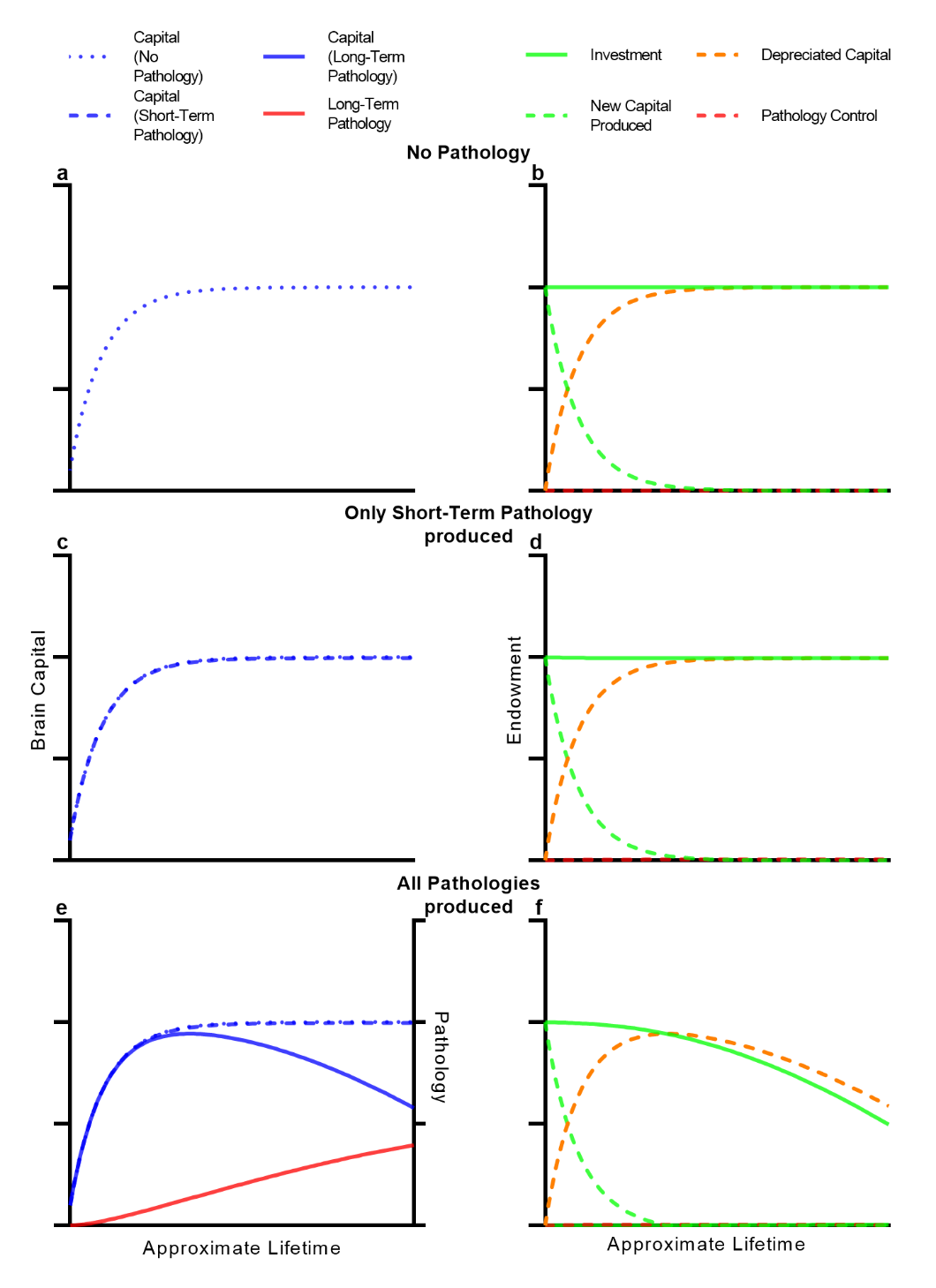


**Supplementary Figure 3. Behavior of the ABC-ND model**

**Supplementary Figure 3. Behavior of the ABC-ND model. a**, Representative simulation of the evolution of brain capital over the lifespan in the absence of either short-term or long-term pathology. **b**, Again, investment (solid green) gradually distributes from producing new brain capital (dotted green) to maintaining depreciated brain capital (dotted orange), driving the plateau in total brain capital. **c**, In the presence of short-term pathology, brain capital again plateaus. **d**, Investment into new brain capital declines only marginally faster with the additional need for pathology control (dotted red) because ABC-ND models the production of only a single form of pathology. **e**, When long-term pathology (solid red) is produced, it accumulates over the lifespan driving a decline in brain capital. **f**, Resources available for investment into capital progressively decline due to neurodegeneration and are outstripped by brain capital depreciation. This difference is the basis for loss of brain capital with neurodegenerative aging.


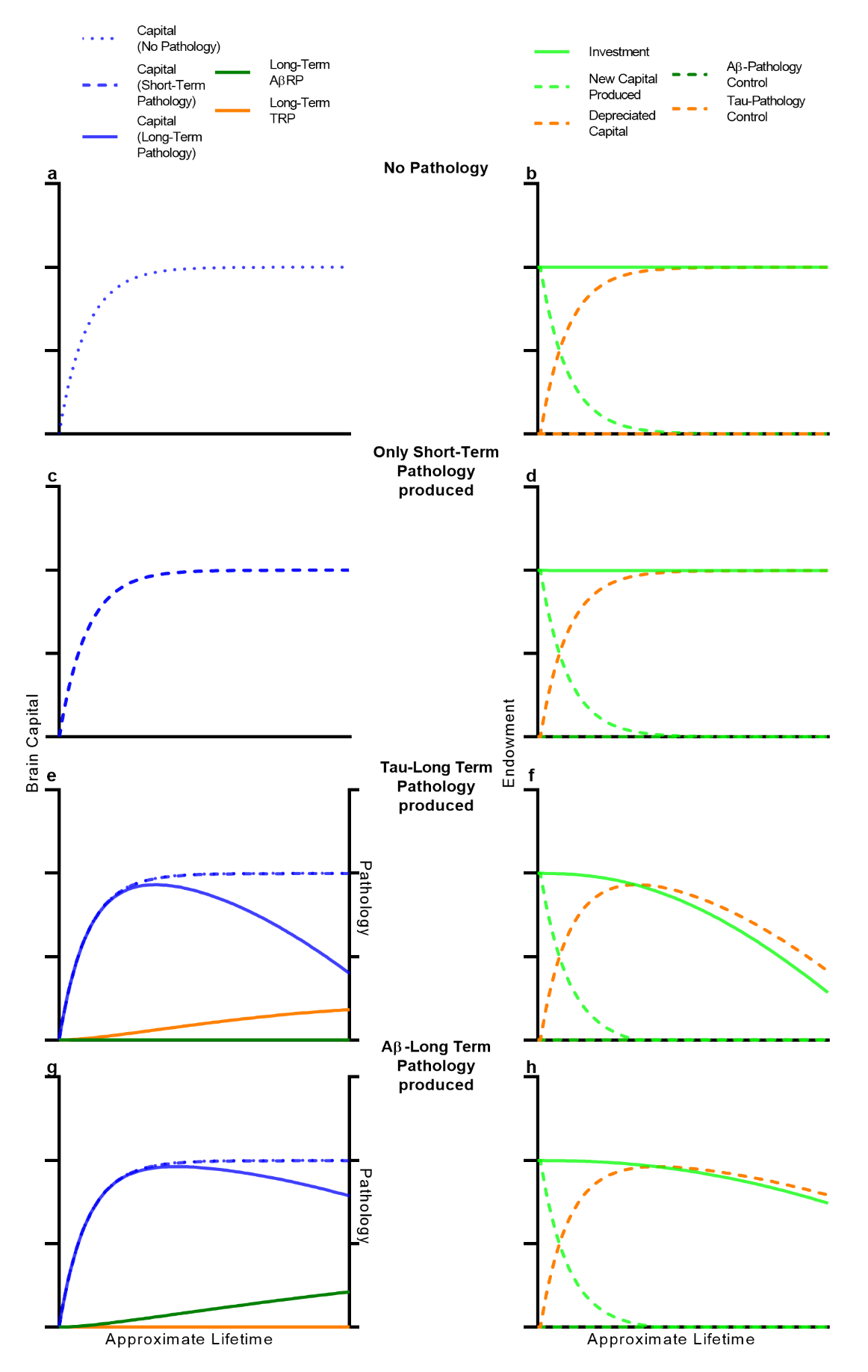


**Supplementary Figure 4. Behavior of the ABC-AD model**

**Supplementary Figure 4. Behavior of the ABC-AD model. a**, Representative simulation of the evolution of brain capital over the lifespan in the absence of either short-term or long-term pathology. **b**, Again, investment (solid green) gradually distributes from producing new brain capital (dotted green) to maintaining depreciated brain capital (dotted orange), driving the plateau in total brain capital. **c**, In the presence of both forms of short-term pathology, brain capital again plateaus. **d**, Investment into new brain capital declines only marginally impacted by the additional need for pathology control (dotted red) because ABC-AD represents the production of only two forms of pathology. **e**, When long-term TRP (orange) is produced alone, it accumulates over the lifespan, driving a progressive decline in brain capital. **f**, Resources available for investment into capital decline due to neurodegeneration and are outstripped by capital depreciation. This difference drives the loss of brain capital due to the accumulation of tau pathology as part of neurodegenerative aging. These trends are also observed with AβRP (**g-h**), although to a lesser extent because the impact of TRP is modeled as greater than AβRP in this simulation.


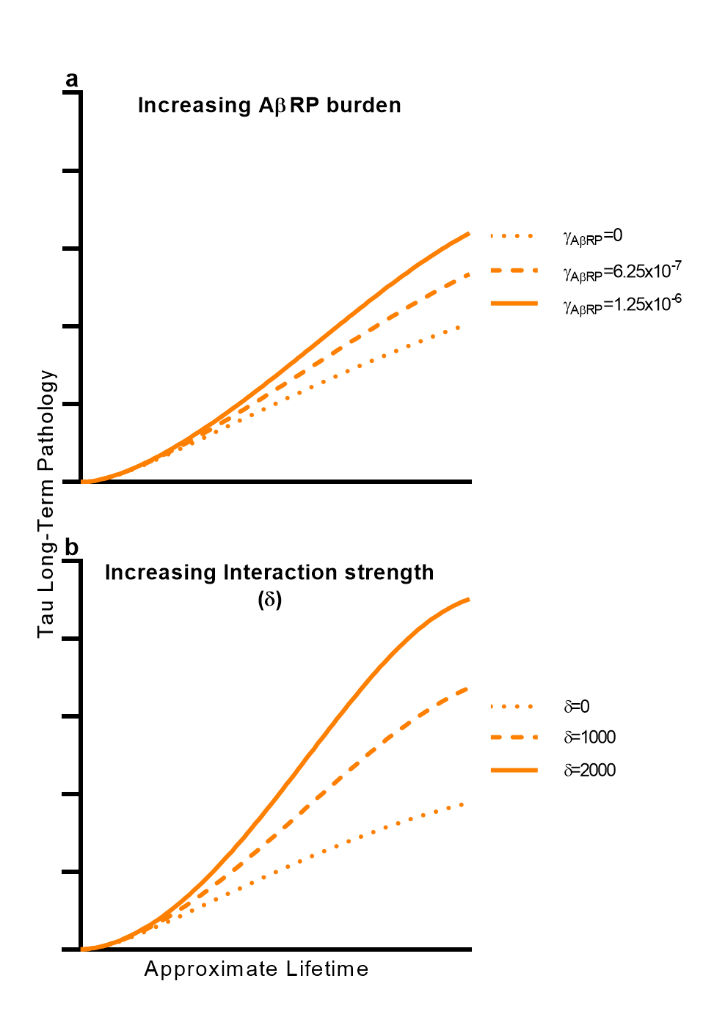


**Supplementary Figure 5. The impact of amyloid-tau interaction**

**Supplementary Figure 5. The impact of amyloid-tau interaction**. **a**, The total production of long-term TRP over the lifespan is accelerated by increased rates of production of AβRP (solid and dotted orange lines). **b**, The total production of long-term TRP over the lifespan is accelerated by an increased strength of interaction between amyloid and tau (represented by the parameter δ).


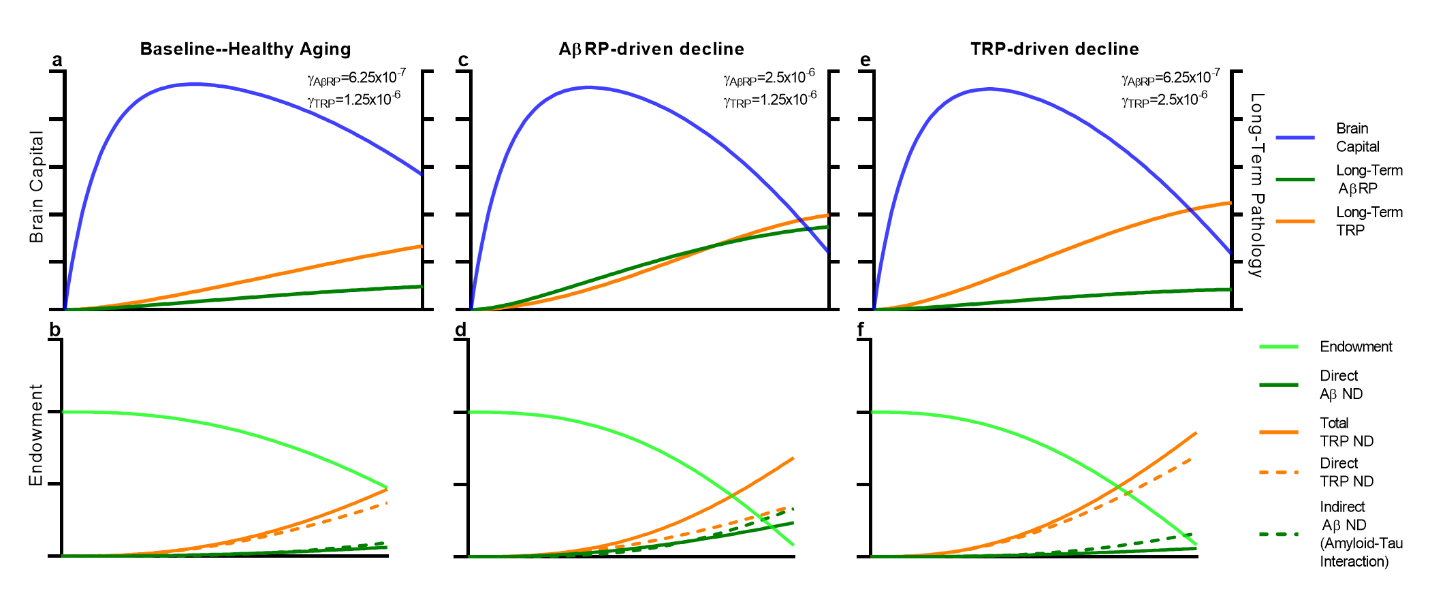


**Supplementary Figure 6. Contributions of Amyloid and Tau to Alzheimer pathogenesis**

**Supplementary Figure 6. Contributions of Amyloid and Tau to Alzheimer pathogenesis.** **a**, Simulation of the lifespan in ABC-AD in the presence of both AβRP (green) and TRP (orange) with minimal pathology accumulation and brain capital (blue) decline. **b**, Decline in endowment (solid green) due to neurodegeneration (ND) is mediated directly by AβRP (dark green) and by TRP (solid orange), which has both a direct component (dotted orange) reflecting the inherent production of TRP and an AβRP-indirect component (dotted green) representing acceleration of TRP due to amyloid-tau interaction. **c**, When AβRP accumulates at a greater rate, TRP is accelerated from baseline and severe capital decline is observed. **d**, TRP contributes most significantly to neurodegeneration although damage to the brain is also mediated directly and indirectly by AβRP. **e**, When TRP is over-produced in a conceivable scenario for tauopathy, it accumulates over the lifespan, driving a progressive decline in brain capital. **f**, In tauopathy, TRP contributes most significantly to neurodegeneration independent of AβRP.


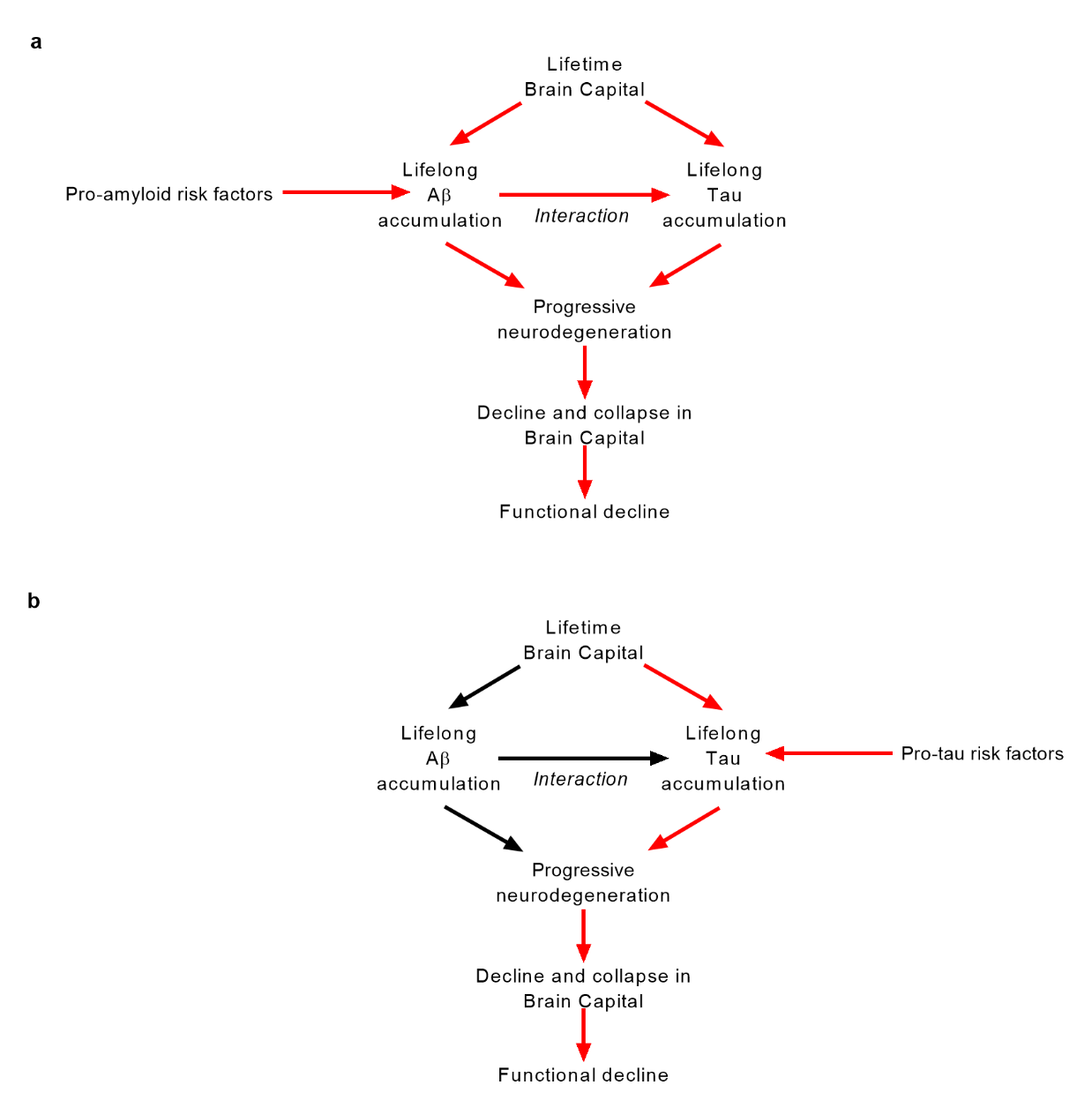


**Supplementary Figure 7. A basic framework for the pathogenesis underlying Alzheimer Disease**

**Supplementary Figure 7. A basic framework for the pathogenesis underlying Alzheimer Disease. a,** Genetic and environmental risk factors that promote the formation of amyloid pathology cause it to accumulate leading to neurodegeneration directly. Perhaps more importantly, this engages amyloid-tau interaction and thus accelerates the accumulation of tau and its impact on neurodegeneration (red arrows). **b,** Genetic and environmental risk factors that promote the formation of tau pathology cause it to accumulate leading to neurodegeneration directly (red arrows) as tauopathy independent of amyloid.


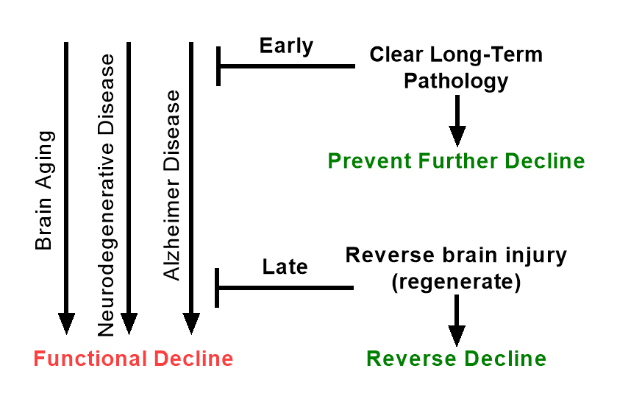


**Supplementary Figure 8. A general strategy for therapeutic intervention**

**Supplementary Figure 8. A general strategy for therapeutic intervention.** Therapies that physically clear the offending brain pathologies have the potential to stop disease progression, especially at an early stage when brain capital and thus function is still relatively preserved. With better prediction and diagnosis, this approach could be developed and implemented as a means to prevent severe decline. Ultimately, the development of neuroregenerative approaches that replete the brain’s non-renewable resources could allow for the reversal of decline in brain capital even at a late stage in disease.
